# Immunological Drivers in Graves' Disease: NK Cells as a Master Switcher

**DOI:** 10.3389/fendo.2020.00406

**Published:** 2020-07-17

**Authors:** Daniela Gallo, Eliana Piantanida, Matteo Gallazzi, Luigi Bartalena, Maria Laura Tanda, Antonino Bruno, Lorenzo Mortara

**Affiliations:** ^1^Endocrine Unit, Department of Medicine and Surgery, University of Insubria, ASST dei Sette Laghi, Varese, Italy; ^2^Immunology and General Pathology Laboratory, Department of Biotechnology and Life Sciences, University of Insubria, Varese, Italy; ^3^IRCCS MultiMedica, Milan, Italy

**Keywords:** natural killer cells, Graves' disease, autoimmunity, hyperthyroidism, inflammation

## Abstract

Graves' disease (GD) is a common autoimmune cause of hyperthyroidism, which is eventually related to the generation of IgG antibodies stimulating the thyrotropin receptor. Clinical manifestations of the disease reflect hyperstimulation of the gland, causing thyrocyte hyperplasia (goiter) and excessive thyroid hormone synthesis (hyperthyroidism). The above clinical manifestations are preceded by still partially unraveled pathogenic actions governed by the induction of aberrant phenotype/functions of immune cells. In this review article we investigated the potential contribution of natural killer (NK) cells, based on literature analysis, to discuss the bidirectional interplay with thyroid hormones (TH) in GD progression. We analyzed cellular and molecular NK-cell associated mechanisms potentially impacting on GD, in a view of identification of the main NK-cell subset with highest immunoregulatory role.

The autoimmune thyroid disorder, known as Graves' disease (GD), is the most frequent cause of hyperthyroidism in iodine sufficient areas (1). Production of autoantibodies against the TSH-receptor (TRAb) represents the ultimate step for disease progression (2). Therefore, identification of the major drivers involved in triggering and progression of the disease, still represents an unmet need (1). There is a large consensus that identification of all potential factors involved in the pathogenesis of GD might favor the development of a more efficient treatment strategy, as well as of prevention approaches (3). This would be of paramount importance in view of the current lack of an effective pharmacological therapy for GD (4–6).

Natural killer (NK) cells, whose has been initially defined in virus clearance and defense against tumors, represent a highly heterogenous cell population. More recently, they have been shown to be involved in autoimmune disorders with both pathogenic and regulatory roles (7). While it is widely accepted that abnormalities in the adaptive immune response underpin autoreactivity and autoimmune diseases, it is also clear that other effector cells within the innate immunity compartment can act as relevant players. The major aim of this narrative review was to discuss the potential involvement of NK cells in the pathogenesis of Graves' disease and to speculate on potential future treatment/prevention strategies, based on NK cells as a target and/or as a tool for therapy.

## Current Understanding of the Pathogenesis of Graves' Disease

Although GD can occur at any age and in both genders, it is more frequently observed in women in the 4–5th decade of life (1). The ultimate event is the continuous activation of the TSH-R on thyroid follicular cells by TRAb (8, 9). This dysregulated and continuous thyroid stimulation causes hyperthyroidism and, frequently, thyroid enlargement (goiter) (10, 11). As for other autoimmune disorders, GD likely results from the breakdown in the immune tolerance mechanisms, both at systemic (peripheral blood) and local (tissue) levels (8, 9). Failure of T regulatory (T reg) cell activity, proliferation of autoreactive T and B cells, and enhanced presentation of TSH-R (due to increased HLA-D affinity for TSH-R, more immunogenic TSH-R haplotype, or increased exposure of TSH-R peptide) drive the development of the disease (12, 13). Interestingly, TRAb has been detected in serum only shortly before diagnosis of GD (8).

Studies of dizygotic and monozygotic twins showed that genetic predisposition plays a relevant role in the development of GD (14, 15). Genetic risk factors for GD include multiple susceptibility genes, such as some HLA haplotypes (e.g., HLA DRB1^*^3, DQA1^*^5, DQB1^*^2), polymorphisms of genes involved in T and B cells regulation [Cytotoxic T-Lymphocyte Antigen 4 (CTLA4), CD40, Protein tyrosine phosphatase non-receptor type 22 (PTPN22), the B cell survival factor (BAFF), Fas-ligand or CD95 and CD3γ], T reg cell functions (FOXp3), and polymorphisms of genes encoding for thyroid peptides (variants of thyroglobulin or TSH-R) (12, 16–21). Recently, a single polymorphism in tumor necrosis factor α (TNFα) gene (rs1800629) was correlated with an increased risk to develop GD (22). GD is a heterogeneous disease, resulting from the combination of various and different gene polymorphisms, actually detectable by pooled genome wide association study (21–25). This would explain the weak overall size effect for genetic markers in genome-wide association studies (16, 21). Precipitating factors, probably inducing epigenetic changes include sex hormones, pregnancy, cigarette smoking, stress, infection, iodine, and other potential environmental factors (17, 26–33).

GD has been historically considered a T helper (Th)2-skewed disorder (34). This was supported by the starring role of B cells and by the features of Th cells infiltrating the thyroid gland, which are T cell clones specific for the TSH-R and mainly harbor Th2 cytokines (34, 35). More recently, Nagayama et al. demonstrated that the induction of immune shifting toward a Th2 phenotype in a GD mouse model was associated with a decrease, rather than an increase, in TRAb synthesis (36). This indirectly suggested a Th1 priority role in the induction of GD (35). In keeping with these findings, several studies showed that thyrostatic treatment with antithyroid drugs progressively induced transition from Th1 to Th2 predominance (37). As elegantly demonstrated by Rapaport and McLachlan, the fact that TRAb antibodies belong to the subclass of IgG, might explain the Th1-Th2 cytokine bias (38). Indeed, different IgG subclasses might coexist in several diseases and could additionally contribute to the pathogenic mechanisms (35–43). While early stage of the humoral immune response involves Th1 cytokines (e.g., IFN [interferon] γ), the prolonged immunization depends on IgG4 antibodies, driven by Th2 cytokines (e.g., interleukin [IL]-4) (39, 40). During a first phase, antigen presenting cells (APCs) and B cells-derived cytokines (IFNγ and TNFα) stimulate thyrocytes to secrete several chemokines, including C-X-C chemokine 10 that can recruit Th cells. Th cells interact with B cells to produce antibodies (1). Finally, intrathyroidal Th2 cells inhibit Th1 responses through the secretion of IL-10, IL-5, and IL-4 (38–46), thus preventing destruction of the thyroid gland, at variance with Hashimoto's thyroiditis. At this stage, thyroid gland might be protected from destruction both by inhibition of macrophages (from Th2 cytokines) and by upregulation of anti-apoptotic mechanisms (BCL-XL)/downregulation of Fas-Fas-ligand interaction (44, 45). Concomitantly, the increased Th2 response leads to an increased production of antibodies.

## Natural Killer Cells and Their Role in Autoimmunity

**Figure 1:**
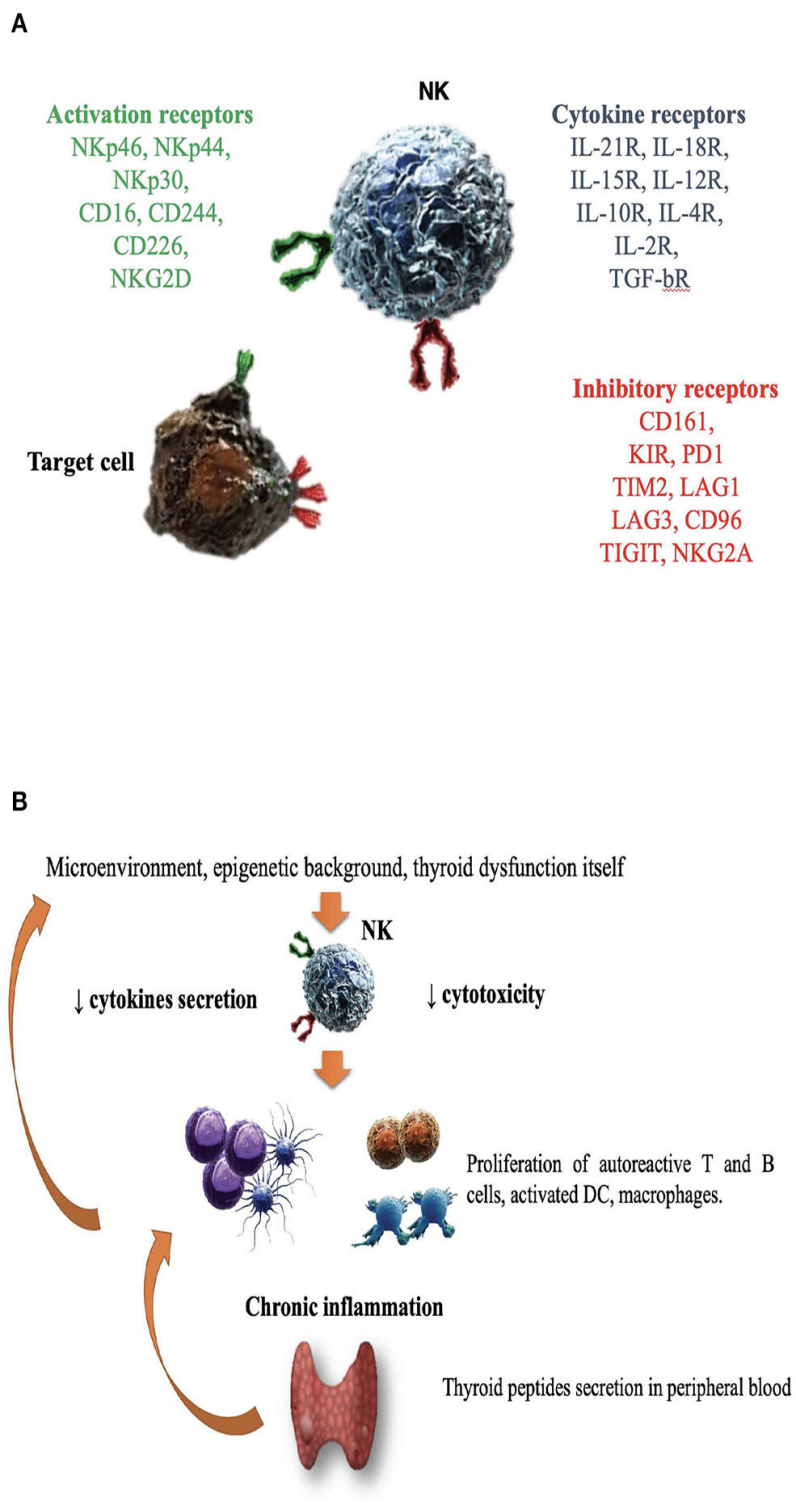
The role of natural killer cells in the pathogenesis of Graves' disease. **(A)** Enumeration of activating/inhibitory receptors and cytokines receptors, whose signals determined NK cells activity in health and disease. CD, cluster of differentiation; CD16, Fc receptor; CD244, non MHC biding receptor acting as costimulatory ligand for NK cells; CD69, early expressed after NK cell activation; CD96, interacts with nectin and nectin-like proteins; CD161, recognizes the human NKR-P1A antigen; KIR, killer cell immunoglobulin like receptor; LAG1 and LAG3, lymphocyte activation gene 1 and 3; NKp30, NKp44, NKp46, the natural cytotoxic receptors (NCR); NKG2A and NKG2D, natural killer group 2A and 2D; TIGIT, T cell immunoglobulin and ITIM domain; IL (interleukin)-21/18/15/10/12/4/2 R (receptor); TGF-bR, TGF beta receptor family; PD1, programmed cell death protein 1; TIM2, T-cell immunoglobulin and mucin-containing domain 2; **(B)** Several factors including microenvironment, cytokines milieu, epigenetic background and hyperthyroidism itself might impair NK protective activity. DC, dendritic cells; NK, natural killer cells.

NK cells are large granular lymphocytes (LGL), recently classified as a subset of innate lymphoid cells (47). They are classically distinguished from the other mononuclear cells due to the expression of CD56, a molecule mediating homotypic adhesion, and null expression of CD3 (48). Additionally, based on the density of CD16 espression (a low-affinity receptor for the Fc portion of immunoglobulin G) and CD56 surface markers, NK cells could be further distinguished in two major subsets: CD56^bright^CD16^dim/−^ and CD56^dim^CD16^+^cells (49– 51). According to a well-supported theory, NK cell precursors leave the bone marrow, transit through peripheral blood and reach the lymph nodes, where, under the influence of cytokines produced by stromal matrix, they differentiate into CD56^+^CD16^−^ (49– 53). Maturation process is characterized by the down-regulation of CD56 and the acquisition of CD16 markers, as well as of “killer cell immunoglobulin-like receptors” (KIRs), getting the features of CD56^dim^CD16^+^cells (50, 52–54). Therefore, CD56^dim^CD16^+^ NKs show high potential of cytotoxicity, due to the high content of cytolytic granules (containing perforin and granzyme), the high expression of KIRs, ILT2 (immunologlobulin-like transcript 2), and CD16 itself (51, 53). Conversely, CD56^bright^CD16^dim/−^ are more immature cells, characterized by poor cytotoxic ability, high expression of inhibitory receptors (such as NKG2A), high ability to proliferate in response to IL-2 and elevated production of several cytokines, such as IFNγ, TNFα, granulocyte–macrophage colony-stimulating factor, IL-10 and IL-13, depending on the conditions of stimulation (51, 55–58). It is the balance between inhibitory and activating signals, deriving from non-rearranged surface receptors, to dictate whether or not NK cells will kill target cells, engaged during their “patrolling” action (Figure 1A). Inhibitory receptors such as NKG2A, CD161, and inhibitory KIRs prevented the killing of normal cells, through the recognition of “self” molecules belonging to MHC class I. Thus, according to the “missing self-hypothesis,” NK cells recognize and attack target cells presenting low or aberrant MHC class I molecules (59). Furthermore, activating receptors, such as the natural cytotoxic receptors (NKp44, NKp46, NKp30), CD69, activating C-type lectin-like receptors (as the natural killer group 2D receptor) and activating KIRs recognize ligands induced on stressed cells (infected/overactive/transformed cells) and stimulate NK cells activation.

With the advent of the single cell technologies, coupled with RNA sequencing, it has been observed that NK cell heterogeneity, in term of subsets, is more complex (according to the different surface antigens and cytokine milieu) (60). This is not only a gene-restricted but also an environmental (re)-directed process (61–64). Modeling T cell classification, in humans, NK cells could be divided at least in two sets: “NK1,” characterized by the production of IFNγ and the regulatory “NK2” cells (65, 66). The polarization to NK2 phenotype depends on high IL4 levels and is characterized by the high production of “type 2” cytokines (i.g., IL-5, IL-10, and IL-13), the high expression of cytokines receptors and of NKG2A surface marker.

Considering their role in defense against viruses and that viral triggers are often involved in the initiation of several immune disorders, NK cells have been investigated for their role in autoimmunity (65–68). Indeed, CD56^bright^ NK cells may orchestrate the overall immune process, influencing both innate and adaptive immune cells, through the integration of signals from numerous activating and inhibitory receptors. Due to the high plasticity and interaction with other immune and stromal cells, CD56^bright^ NK cells acquire a regulatory role (65–69). In this context, a third subset, called “NK reg” has therefore been suggested and defined, according to surface inducible or constitutive markers such as CD117 (65–73). However, the available studies provided conflicting results, since, under some circumstances, NKs play a protective role, while in others they have been blamed to be pathogenic (7, 62, 68, 69). Likely, their action is correlated to the type of cell becoming the target of attack. In case of whether acquired or inherited dysfunctions, NK cells might participate into the destruction of non-transformed, healthy cells as the first step of the autoimmune process. Conversely, if targets are autoreactive T cells, dendritic cells (DC) or pro-inflammatory macrophages, NKs might act as regulators, dampening the inflammatory process (65, 69–73). Interestingly, NK cell regulatory activity has been demonstrated in several autoimmune diseases, such as multiple sclerosis (MS), experimental colitis or encephalitis (EE) and arthritis (RA), by different strategies such as cytokine release, interaction with ligands of the receptors NKG2D, NKG2A, NKp46 or perforin-mediated T cell death (63, 72, 73). In a mouse model, Ehelers and co-workers demonstrated that high levels of IL-18, which are found in Th1-skewed autoimmune process, induced the expression of CD117 on NKs which, in turn, became able to suppress CD8^+^T cells (73). In other experiments, CD56^bright^ NK suppressed autologous CD4^+^ T cells proliferation through the expression of NKp30 and NKp46, granzyme B releasing and immunosuppressive molecule adenosine (72, 73). In experimental models of autoimmune EE, the inhibitory role of NKs on the T effectors proliferation, as well as a direct cytotoxic effect on autoreactive specific T cells, were shown (74). Likewise, Takahashi et al. demonstrated, in MS patients, that CD56^bright^ NK could favor clinical remission, by suppressing the production of IFNγ, by specific autoreactive T effectors and secreting IL-5 (57, 69, 75). Laroni et al. observed that CD56^bright^ NK cells had reduced ability to kill T-cells in MS patients, compared to healthy controls, possibly due to an increased expression of NKG2A (69, 76). Thus, impaired cytotoxicity or the inability to secrete cytolytic granules have been correlated to the escape of proinflammatory cells (both T and B lymphocytes, DC and macrophages) from regulatory mechanisms of controls (77). In other cases, such as RA, loss of NK tolerance (due to decreased inhibitory signals or inappropriate stimulation of activating signals) might favor the development of autoimmune diseases. Different mechanisms have been blamed, such as the presence of antilymphocyte antibodies (78). In other disorders, such as myasthenia gravis and EE, NK cells seem to facilitate initiation and progression of autoimmunity (67, 68). Besides differences in the strains and models used, several factors may influence the specific, and even contradictory, actions of NK cells. Their ability to adapt to different stimuli and different anatomical localization may play an important role. Microenvironment itself may influence NK functions, such as migration and tissue retentions, as it emerged in the complex interaction with DC, influenced by density, maturation state and phenotype of this population (68). Epigenetic modifications strongly influence NK cells all along their life, from development to regulation and differentiation of effector functions (79–81). Epigenetic remodeling, acquired through immunological experiences, might modulate NK functions (61). For instance, gene expression of several genes (including KIRs) is regulated by DNA methylation (hypomethylation or hypermethylation) of their promoters. The interindividual genetic variability in the receptor repertoire, especially of the highly polymorphic KIR gene, influence the recognition of target cells (80). KIRs polymorphisms might influence the engagement with HLA molecules and, as counterpart, functional interaction between co-inherited KIRs (especially inhibitory KIRs) and HLA progressively influence NK education (81). Besides KIRs, other receptors such as NKG2A are involved in NK education (61).

## The Link Between Leukocytes and Thyroid Hormones

A possible link between THs and the immune system was already suggested more than 40 years ago, by the discovery that Staphylococcus-stimulated lymphocytes might *de novo* synthesize a TSH-like substance (immunoreactive TSH, i-TSH), similar to the pituitary-released form and possibly involved in autoimmune thyroid disorders (AITD) (82). Further experiments progressively demonstrated that bone marrow hematopoietic cells, lymphocytes, DC and even intestinal epithelial cells, could synthesize TSH (83). The role of extra-pituitary TSH remains to be clarified. It was speculated that, as pituitary TSH, i-TSH might stimulate the synthesis of TH, which, in turn, might influence the immune system (indirect effect). Several papers showed that immune cells harbor essential elements required for THs metabolism and action. For example, both neutrophils and DC express T3 (the active form of TH) transporters (MCT10 in human) and type 2 and 3 deiodinases (involved in THs synthesis) (84–86). Indeed, it has been widely demonstrated that THs interact with hematopoietic cells (85–90) at different levels. T3 might affect target immune cells by binding both to nuclear receptors (thyroid hormones receptors TRα and TRβ) and membrane receptors (86–90). For example, TH and especially T3 can influence maturation of DCs (84, 85). DC phenotype was studied in thyroidectomized patients before and after levothyroxine supplementation, showing that THs induce an increase in DCs number and influence their functions (91). A research group from Cordoba demonstrated that T3 induce DCs activation through Akt and NF-kB pathways, driving the immune response toward a Th1 phenotype (92, 93). Further support to the regulatory role of TH came from experiments showed that daily administration of T4 was followed by the complete restoration of the immune competence in thyroidectomized mice (94). Furthermore, T4 treatment in mice enhanced the NKs cytotoxic activity against classical target cells, amplifying their responsiveness to cytokines and modulating NK metabolic properties (95). Some years later, Provinciali et al. demonstrated that, after T4 pre-treatment, the peak of NK cytotoxic activity was achieved using half the optimal IFNγ concentration (96). Additional experiments strengthen the hypothesis of a paracrine TSH-pathway (97–99). TSH-R is expressed on myeloid and lymphoid cells (100, 101). By its stimulation, TSH (both the immune and the pituitary released forms) may act as a cytokine-like regulatory molecule and induce the secretion of several cytokines, such as TNFα (102, 103). *In vitro* studies showed that TSH, combined to classical cytokines (as IL-2, IL-12, IL-1β), acts as co-stimulus improving lymphocytes and NKs proliferative response to even low dose of mitogens (103, 104). Todd et al. demonstrated that TSH was able to enhance the expression of MHC class II in thyroid cells treated with IFNγ (105). Accordingly, Dorshkind et al. demonstrated that THs induce the synthesis of cytokines and the expression of IL-2 receptor in NK cells (106). Indeed, while both T3 and FT4 boosted the IFNγ response in mice (107, 108), T4 amplified both IFNγ and IL-2 (96).

Based on the bidirectional relationship between TH and the immune system (96), Kmiec et al. postulated that in the elderly the reduction of TH with aging might be involved in the impairment of NK activity by T3 administration; they found a direct correlation between serum T3 levels and NK activity, in spite of conserved proportion of circulating NK cells (109, 110). Indeed, NK cell activity was selectively improved by T3 administration in those subjects having T3 levels in the slower range.

## Natural Killer Cells and Graves' Disease

From a mutual perspective, thyroid function might orchestrate the immune response and, conversely, dysfunction of the immune system might favor the development of thyroid disorders. Several studies investigated the potential contribution of NKs in the development and/or progression of GD, but results are still inconclusive and sometimes conflicting. Table 1 reports the available data on this issue (111–123). Researchers from Osaka University observed that the total percentage of LGL, including NK-like cells, was decreased in untreated GD patients compared to euthyroid GD patients on antithyroid drug therapy and to controls; in addition, the proportion of LGL was inversely correlated to T4 and T3 levels (110–112, 123). Thus, while normal THs levels are crucial to maintain an adequate activity of the immune system, supraphysiological THs levels exerted a detrimental effect, mimicking starvation, and increased cortisol secretion (121, 124–126). Immunocomplexes able to suppress NK cell activity, as in other autoimmune disorders (e.g., RA), were considered as a possible cause of this phenomenon (76, 127). According to a different hypothesis, the decrease of NK cells might be the primary immunological abnormality in the pathogenesis of GD (111, 127).

**Table 1:** Summary of studies investigating the role of natural killer cells in Graves' disease.

**References**	**Subjects**	**Study objects**	**Methods**	**Outcome**
Amino et al. (111)	GD (16 untreated GD + 11 hyperGD under ATD + 3 euGD under ATD + 4 remission GD) vs. 43 controls vs. 14 HT	K lymphs	Peripheral blood samples	**↓** K lymphs in hyperGD than controls **↓** plaque forming K lymphs in hyperGD than controls No differences in K lymphs comparing euGD to controls.
Iwatani et al. (112)	GD (12 hyperGD + 5 euGD) vs. HT (17 euHT + 4 hypoHT) vs. 55 controls	LGL	Peripheral blood samples	LGL **↓↑**FT4, FT3 in hyperGD **↓**LGL in hyperGD compared to other groups
Stein-Streilein et al. (113)	Mice fed with T4 vs. hypothyroid (due to ATD) vs. euthyroid mice	NK release of lytic factors	Blood, spleen and lung samples after 2 and 6 w	**↓**lytic molecules release in thyrotoxic mice
Papic et al. (114)	22 untreated GD vs. 18 hyperthyroxinemic for T4 treatment	cNK number and activity	Peripheral blood samples Release assay for NK cytotoxicity against K562	**↓**cytotoxicity in hyperthyroidism (both groups) **↓**ability of IL-2 chance to enhance NK activity in GD
Wang et al. (115)	GD (33 untreated GD + 19 euGD under ATD + 6 euGD after ATD withdrawal) vs. 43 controls	cNK number, cytotoxicity	Peripheral blood samples Release assay for NK cytotoxicity against K562	No differences in cNK number in GD compared to controls. **↓**cytotoxicity in untreated GD or during ATD treatment vs. euGD
Pedersen et al. (116)	20 untreated GD vs. 11 HT vs. 10 non-toxic goiter vs. 22 controls	cNK number, cytotoxicity	Co-culture with IL-2, IFN, indomethacin Release assay for NK cytotoxicity against K562	No differences in cNK number and activity in AITD vs. controls
Lee et al. (117)	18 untreated GD vs. 18 controls	cNK cytotoxicity	Co-culture with T4 Release assay for NK cytotoxicity against K562	No differences in cNK activity in GD vs. controls**↑** cytotoxicity with T4 in controls but not in GD
Hidaka et al. (118)	25 untreated GD vs. 18 HT vs. 22 postpartum AITD vs. 61 controls	cNK cytotoxicity	Peripheral blood samples Release assay for NK cytotoxicity against K562	**↑**cytotoxicity in GD vs. other groups
Aust et al. (119)	10 GD	tNK and cNK number	Thyroid tissues and peripheral blood samples	tNK**↑↑** AbTPO
Wenzel et al. (120)	40 GD vs. 26 HT vs. 32 controls	cNK cytotoxicity	Peripheral blood samples Release assay for NK cytotoxicity against K562	**↓** cytotoxicity in untreated/under ATD GD vs. controls
Solerte et al. (121)	13 untreated GD vs. 11 hypoHT vs. 15 controls	Functional studies	cNK were incubated with IL-2, TGF-β and DHEAS Release assay for NK cytotoxicity against K562 Cytokine secretion	**↓**cytotoxicity induced by IL2 e TGF-β in GD and HT **↓**spontaneous and IL2 induced TNFα release
Dastmalchi et al. (80)	8 untreated GD vs. 176 controls	KIR genes and related HLA polymorphisms	Peripheral blood samples PCR-SSP	No evident correlations
Zhang et al. (122)	28 untreated GD vs. 23 controls	Functional and phenotypic studies	Peripheral blood samples	**↓**cytotoxicity in GD vs. controls **↓**NKG2D^+^, NKG2C^+^, NKp30^+^, NKG2A^+^ NK in GD vs. controls **↓**IFNγ in GD vs. controls NKG2A^+^ NK **↓↑**TRAb NKG2D^+^ NK **↓↑**TH

*AITD, autoimmune thyroid disorders; ATD, antithyoid drugs; cNK, circulating NK cells; eu, euthyroidism; d, days; HT, Hashimoto's thyroiditis; IFN-γ, interferon γ; K lymphs, killer lymphocytes; LGL, large granular lymphocytes; NKG2 A/C/D, natural killer group 2D, belonging to C-type lectin like receptors; PCR-SSP, polymerase chain reaction sequence-specific primer directed method; tNK, tissutal (intrathyroidal) NK cells; TH, thyroid hormones; w, weeks; **↑** enhance; **↓** depress; **↑↑** direct correlation; **↓↑** inverse correlation*.

Solerte et al. reported that both spontaneous and IL-2/IFNβ-modulated NK cells cytotoxicity (NKCC), as well as spontaneous and IL-2 induced TNFα release were decreased in NK cells from 13 GD patients compared to 15 controls (121). Both cytokines secretion and cytotoxicity were promptly normalized by co-incubating NKs with DHEAS (dehydroepiandrosterone sulfate), supporting the concept of a concomitant effect of other endocrine axes (121, 128, 129). Studies from the University of Miami comparing thyrotoxic mice (due to levothyroxine treatment) to euthyroid or hypothyroid (due to antithyroid drug treatment) control mice observed a reduced secretion of cytolytic granules (113). Similar results were obtained from the same group in humans (see Table 1) (114), with a reduction in cytotoxicity, studied by release assay for NK cell cytotoxicity against K562 tumor target cells.

Considering that NK cell activity is affected by age (130), a study compared NKCC in AITD patients with age and gender-matched healthy controls, demonstrating an impaired NK cell activity in AITD (120). As previously outlined, the integration of activating and inhibitory signals from NK surface regulates NK cells effector functions, such as cytokine secretion and NKCC. In a study of 28 newly onset GD patients, Zhang et al. observed a reduction of NK cells expressing both activating (NKG2D, NKG2C, NKp30) and inhibitory receptors (NKG2A) compared to matched healthy controls (122). Additionally, NKG2A^+^ NKs were inversely related to TRAb levels, while NKG2D^+^ NKs were inversely related to serum free T4 levels (122), supporting the role of dysfunctional NK cells. Figure 1B illustrates the hypothesis that in case of dysfunctional impairment, NK cells lose their ability to protect from the development of GD. Other studies (115, 117, 119, 120, 122, 131, 132), with some exceptions (116, 118) generally agreed on the impairment of NK activity in GD and reported that restoration of euthyroidism by antithyroid drug treatment (especially propylthiouracil) could improve NK functionality (133, 134).

## Conclusion and Future Perspectives

It is now clear the immune system, both the innate and the adaptive components, are crucial host-related orchestrators of disorder induction/insurgence and progression. Alterations of immune cell phenotype and functions, as a consequence of chronic inflammation, are shared features between cancers, cardiovascular, neurological, and autoimmune diseases. In the new era of immunotherapy, most of the efforts are addressed to cancer, as supported by the vast literature and clinical trials (135). This rapidly developing field suggests the same attention should be dedicated also to autoimmunity, that still requires a better understanding of the cellular and molecular events occurring during autoimmune disorders, including GD. Unveiling these mechanisms and events is required to identify new immunological cellular biomarkers, trace disease progression, and design new targeted therapeutic strategies for autoimmunity. In this scenario, re-education/manipulation of NK cells appear as a promising strategy, as confirmed by the growing interest in CAR-NK cells (136).

## Author Contributions

DG, LM, EP, and AB conceived the manuscript. All the authors took part in manuscript writing and editing. EP, LB, LM, and AB supervised the final version of the manuscript.

## Conflict of Interest

The authors declare that the research was conducted in the absence of any commercial or financial relationships that could be construed as a potential conflict of interest.
